# Optimizing Outcomes in Laparoscopic Hysterectomy: An Analysis of Complications, Management Techniques, and Lessons Learned From Our Clinical Experience

**DOI:** 10.7759/cureus.83636

**Published:** 2025-05-07

**Authors:** Sakshi Agarwal, Sangeeta Rai, Lalit Kumar, Anubhuti Gupta

**Affiliations:** 1 Department of Obstetrics and Gynecology, Institute of Medical Sciences, Banaras Hindu University, Varanasi, IND; 2 Department of Urology, Institute of Medical Sciences, Banaras Hindu University, Varanasi, IND

**Keywords:** complications, hysterectomy, hysterosalphingectomy, laparoscopic, total laparoscopic hysterectomy

## Abstract

Background: Laparoscopic hysterectomy has emerged as a preferred surgical option for managing various gynecological conditions due to its minimally invasive nature and favorable outcomes. This study aimed to evaluate the demographics, indications, complications, and recovery outcomes associated with laparoscopic hysterectomy in a cohort of patients.

Methods: A retrospective analysis was conducted on 117 patients who underwent laparoscopic hysterectomy at a single institution. Data were collected regarding patient demographics, surgical indications, intraoperative and postoperative complications, and discharge timing. Statistical analyses were performed to assess the relationships between these variables.

Results: Most patients were between 41 and 50 years (58.1%), with a significant proportion being multiparous (47.9%). The primary indications for surgery included fibroids (42.7%) and abnormal uterine bleeding (35.0%). Intraoperative complications were low, with bladder injury occurring in 2.6% and bowel injury in 0.85% of cases. The conversion rate to open surgery was 5.1%. Postoperatively, complications such as intestinal obstruction, paralytic ileus, peritonitis, and port-site infection were reported in 0.85% of patients each, while 96.6% experienced no complications. Most patients (89.75%) were discharged within 48 hours after surgery.

Conclusion: The findings of this study support the effectiveness and safety of laparoscopic hysterectomy as a surgical option for women with gynecological conditions. Despite some patients requiring extended hospitalization, the overall hospital stay and complication rates were low, reinforcing the viability of laparoscopic techniques in clinical practice. Future research should focus on larger, prospective, or randomized multicenter studies with extended follow-up periods to validate these results and assess long-term outcomes of laparoscopic hysterectomy.

## Introduction

Laparoscopic hysterectomy, a minimally invasive surgical procedure for the removal of the uterus, has become a popular alternative to traditional abdominal and vaginal hysterectomies [1]. Total laparoscopic hysterectomy (TLH) offers benefits such as reduced postoperative pain, improved cosmesis, shorter hospital stays, and faster convalescence, making it an attractive option for patients and surgeons [2,3]. As the utilization of TLH has increased, understanding and managing its associated complications have become crucial for ensuring patient safety and optimizing surgical outcomes. While TLH is generally considered safe and effective, it is not without risks [4].

Different studies show that the complication rate of TLH is up to 10% [3]. A study analyzing complications of TLH found that intraoperative complications included bladder injury (0.09%), bowel injury (0.09%), internal iliac vessel bleeding (0.03%), and conversion to vaginal hysterectomy due to cautery failure (0.03%)1. Postoperative complications included vault bleeding (2.75%), intestinal obstruction (0.06%), paralytic ileus (0.15%), vesicovaginal fistula (0.03%), ureterovaginal fistula (0.03%), and peritonitis (0.03%) [5].

To enhance patient safety and optimize surgical outcomes, it is essential to identify and address variables that contribute to intraoperative complications during TLH. Factors such as the surgeon's experience, patient characteristics (e.g., obesity, previous surgeries), and uterine size can influence the likelihood of complications. A study showed that significant complications occurred in 4.4% of laparoscopic hysterectomies [4]. Understanding these factors and implementing strategies to mitigate their impact is vital for minimizing risks and improving the overall quality of care in TLH [3,6]. This study aimed to comprehensively analyze the intraoperative and postoperative complications associated with TLH over a specified period. The primary objectives are to analyze the intraoperative and postoperative complications of TLH over a period and evaluate proximate variables associated with intraoperative complications of TLH.

## Materials and methods

This retrospective observational study was conducted in the Department of Obstetrics and Gynecology from September 1, 2021, to September 30, 2024. Its primary aim was to analyze the intraoperative and postoperative complications associated with total TLH and evaluate the factors contributing to complications. The secondary objectives were to assess the need for blood transfusion and the duration of hospital stay.

The study population consisted of women undergoing TLH for various benign and pre-malignant gynecological conditions at the Institute of Medical Sciences, Banaras Hindu University, in northern India. Strict inclusion and exclusion criteria were applied to ensure a homogeneous study population. The inclusion criteria encompassed patients undergoing TLH at the specified institution within the study period, those with complete preoperative evaluations and fitness for laparoscopic surgery, and patients willing to provide informed consent. Exclusion criteria included those undergoing hysterectomy via other surgical approaches (e.g., abdominal, vaginal, laparoscopic-assisted vaginal hysterectomy), patients undergoing TLH for emergent indications (e.g., severe hemorrhage, uterine rupture), patients with known pre-existing conditions that significantly increase the risk of complications (e.g., advanced malignancy, severe coagulopathy) and patients with incomplete medical records or who were lost to follow-up.

A total of 117 patients were included in the study. The sample size was determined based on the feasibility of recruiting patients within the study period and the desire for sufficient statistical power to detect meaningful associations between risk factors and complications. Sample size calculations were performed based on an estimated complication rate of 4.4% (Madhvani et al. [7]). Based on this calculation, a sample size of approximately 65 patients would be needed to estimate the complication rate with a 5% margin of error. Since our study included 117 patients, which exceeds the calculated requirement of 65, our sample size appears adequate for estimating the complication rate with a 5% margin of error and 95% confidence level.

Ethical principles and guidelines were used to conduct the study. The Institutional Ethics Committee (IEC) approved the study protocol at the Institute of Medical Sciences, Banaras Hindu University, Varanasi, for review and approval (IMS/IEC/2024/7716). Data were collected retrospectively using a standardized form encompassing patient demographics, preoperative evaluations, surgical data, intraoperative complications, postoperative complications, management of complications, and follow-up data. These details were filled out with the help of the patient’s admission files and discharge papers, and by calling patients telephonically.

The collected data were analyzed using appropriate statistical methods to describe the incidence of complications and to evaluate potential risk factors. Descriptive statistics were used to summarize patient demographics, surgical data, and the frequency of intraoperative and postoperative complications.

Continuous variables were presented as means with standard deviations or medians with interquartile ranges, as appropriate. Categorical variables were presented as frequencies and percentages. The incidence of intraoperative and postoperative complications was calculated as the number of events divided by the total number of TLH cases. The multivariable and regression analyses were done to investigate predictors of major complications of surgeries. Statistical analysis was performed using IBM SPSS Statistics for Windows, Version 23 (Released 2015; IBM Corp., Armonk, New York, United States).

## Results

Table 1 illustrates that most patients undergoing laparoscopic hysterectomy in this study are 46-50 years (31.6%) and 41-45 years (26.5%). The data indicate a decreasing frequency of surgeries among older age groups (51-55 and 56-60), suggesting that younger and middle-aged women are more likely to seek this surgical intervention. Overall, the total number of participants in the study was 117, representing a diverse age range with a clear trend toward younger demographics for laparoscopic hysterectomy procedures. Among the 117 participants, only three patients (2.6%) reported having no children, indicating that most women had some childbirth experience. A significant portion, 48 patients (41.0%), had 1-2 children, while the largest group consisted of 56 patients (47.9%) who had 3-4 children, suggesting that many women undergoing this procedure have had multiple pregnancies. Finally, 10 patients (8.5%) reported having more than four children. Overall, the data reflect a diverse range of reproductive histories among the women in this study.

**Table 1:** Age & parity distribution.

Age group (years)	Frequency	Percent
35-40	30	25.6
41-45	31	26.5
46-50	37	31.6
51-55	13	11.1
56-60	6	5.1
Total	117	100.0
Parity		
No child	3	2.6
1-2 children	48	41.0
3-4 children	56	47.9
More than four children	10	8.5
Total	117	100.0

Out of the total 117 participants, the majority, 63 patients (53.8%), reported having no previous surgeries, indicating a significant portion of women without prior surgical interventions. Among those with previous surgeries, 40 patients (34.2%) had undergone ligation, making it the most common prior procedure in this cohort. Additionally, six patients (5.1%) had experienced two lower-segment cesarean sections (LSCS), while four patients (3.4%) had undergone one LSCS, and another four patients had a history of laparotomy. These data highlight that while many women had no surgical history, a notable number had undergone ligation, reflecting standard reproductive health practices among the study population (Table 2).

**Table 2:** Type of previous surgery and indications of surgeries. LSCS: Lower-segment cesarean section

Type of previous surgery	Frequency	Percent
One LSCS	4	3.4
Two LSCS	6	5.1
Laparotomy	4	3.4
Ligation	40	34.2
No previous surgery	63	53.8
Indication of surgery		
Pelvic inflammatory disease	17	14.5
Endometrial hyperplasia	5	4.3
Abnormal uterine bleeding	41	35.0
Fibroid	50	42.7
Postmenopausal bleeding	4	3.4
Total	117	100.0

Table 2 also outlines the indications for laparoscopic hysterectomy among the 117 patients included in the study. The most common reason for surgery was the presence of fibroids, with 50 patients (42.7%) undergoing the procedure for this condition. Abnormal uterine bleeding was also a significant indication, affecting 41 patients (35.0%). Additionally, pelvic inflammatory disease was noted as the reason for surgery in 17 patients (14.5%), while endometrial hyperplasia and postmenopausal bleeding were less common indications, with five patients (4.3%) and 4 patients (3.4%), respectively. These data highlight that fibroids and abnormal uterine bleeding are the primary reasons prompting laparoscopic hysterectomy in this patient population.

Table 3 presents the distribution of uterus sizes among the study's 117 patients who underwent laparoscopic hysterectomy. Most patients had uterine sizes between 11 and 12 weeks of gestation, with 37 patients (31.6%) falling into this category. This was closely followed by those with 9-10 weeks of uterine size, comprising 32 patients (27.4%). Additionally, 25 patients (21.4%) had uterine sizes ranging from 7 to 8 weeks, while 15 patients (12.8%) had uterine sizes greater than 12 weeks. The smallest group consisted of eight patients (6.8%) with a uterus size of up to six weeks. Overall, the data indicate that most patients had moderately enlarged uteri, which is often associated with conditions requiring surgical intervention.

**Table 3:** Uterus size.

Uterus size (weeks)	Frequency	Percent
Upto 6	8	6.8
7-8	25	21.4
9-10	32	27.4
11-12	37	31.6
>12	15	12.8
Total	117	100.0

Table 4 summarizes the blood transfusion status of the study's 117 patients who underwent laparoscopic hysterectomy. Out of the participants, only 12 patients (10.3%) required a blood transfusion during or after the procedure, indicating a relatively low incidence of this intervention. In contrast, the vast majority of patients, totaling 105 (89.7%), did not require any blood transfusions. These data suggest that laparoscopic hysterectomy is generally associated with minimal blood loss, contributing to a favorable safety profile for this surgical approach.

**Table 4:** Blood transfusion.

Blood transfusion	Frequency	Percent
Yes	12	10.3
No	105	89.7
Total	117	100.0

Table 5 provides an overview of the types of surgical procedures performed on the study's 117 patients who underwent laparoscopic hysterectomy. The most common method was TLH, which was conducted on 88 patients, accounting for 75.2% of the total cases. Additionally, 11 patients (9.4%) underwent total laparoscopic hysterectomy with bilateral salpingo-oophorectomy (TLH-BSO), while 18 patients (15.4%) had a TLH combined with bilateral salpingectomy. These data highlight that TLH is the predominant surgical approach in this cohort, with a smaller proportion of patients requiring additional procedures involving the ovaries and fallopian tubes. It also presents the data on converting laparoscopic procedures to open surgery among the 117 patients included in the study. Out of the participants, only six patients (5.1%) required conversion to open surgery, indicating that most procedures were completed laparoscopically. In contrast, 111 patients (94.9%) had surgeries without conversion. This low conversion rate suggests that laparoscopic hysterectomy was predominantly effective and safe in this patient population, reflecting positively on the surgical techniques employed and the overall management of complications during the procedures.

**Table 5:** Type of surgery and conversion to open surgery.

Type of surgery	Frequency	Percent
Total laparoscopic hysterectomy (TLH)	88	75.2
Total laparoscopic hysterectomy with bilateral salpingo-oophorectomy (TLH-BSO)	11	9.4
Total laparoscopic hysterectomy (TLH) with bilateral salpingectomy	18	15.4
Total	117	100.0
Converted to open surgery		
Yes	6	5.1
No	111	94.9
Total	117	100.0

Table 6 outlines the intraoperative complications experienced by the study's 117 patients who underwent laparoscopic hysterectomy. Most patients, 108 (90.6%), did not experience any complications during the procedure, indicating a high level of safety associated with laparoscopic surgery. Among those who did experience complications, bladder injury was reported in three patients (2.6%), while bowel injury and conversion to vaginal hysterectomy were each noted in one patient (0.85%). Additionally, four patients (3.4%) required conversion to laparotomy due to complications. These six patients required conversion to open surgery. Usually, a drain was not placed after surgery; only two patients (1.7%) had drains placed postoperatively as part of their management. Overall, the data reflect a low incidence of intraoperative complications, reinforcing the effectiveness of laparoscopic techniques in this surgical population. A majority of patients, 113 (96.6%), did not experience any complications following the surgery, indicating a high level of safety associated with the procedure. Among those who did have complications, there was one case each of intestinal obstruction, paralytic ileus, peritonitis, and port-site infection, with each complication affecting one patient (0.85%). These data highlight that while most patients had a smooth recovery, a few experienced specific postoperative issues, reflecting the need for careful monitoring following laparoscopic hysterectomy.

**Table 6:** Intraoperative and postoperative complications.

Complications	Frequency	Percent
Bladder injury	3	2.56
Bowel injury	1	0.85
Conversion to vaginal approach	1	0.85
Conversion to laparotomy	4	3.4
No complication	108	92.30
Total	117	100.0
Postoperative complications		
Intestinal obstruction	1	0.85
Paralytic ileus	1	0.85
Peritonitis	1	0.85
Port-site infection	1	0.85
No complication	113	96.60
Total	117	100.0

Table 7 presents the discharge timing for the study's 117 patients who underwent laparoscopic hysterectomy. Most patients, 105 (89.75%), were discharged within two days post-surgery. In contrast, only 12 patients (10.25%) were discharged on or after the third day following their procedure. This data suggests that most patients experienced a quick recovery.

**Table 7:** Day of discharge.

Day of discharge	Frequency	Percent
1-2 days	105	89.75
More than two days	12	10.25
Total	117	100.0

In Table 8, none of the analyzed factors reached statistical significance. Specifically, age greater than 45 years (p = 0.893), high parity (more than four children, p = 0.320), previous surgical history (p = 0.069), large uterine size (greater than 12 cm, p = 0.867), and the type of procedure (TLH alone or TLH with bilateral salpingo-oophorectomy, p = 0.695 and 0.651, respectively) were not significantly associated with an increased risk of major complications. The odds ratios for these factors were all close to or above 1, except for parity (>4 children), which had an odds ratio above 2, suggesting maximum odds of complications. However, this result was not statistically significant.

**Table 8:** Predictors of major (intraoperative + postoperative + conversion to open procedure and hospital stay) complications following total laparoscopic hysterectomy by multivariable analysis.

Parameters	p-value	Odds ratio	95.0% C.I.for EXP(B)	
			Lower	Upper
Age >45 years	.893	1.099	.277	4.358
Parity (>4 children)	.320	2.330	.440	12.336
Presence of Previous Surgery	.069	.264	.063	1.109
Uterus size (>12 cm)	.867	1.206	.134	10.822
TLH	.695	1.403	.257	7.653
TLH-BSO	.651	1.852	.128	26.685

## Discussion

In the current study, most patients were between 46 and 50 (31.6%) and 41 and 45 (26.5%). This aligns with a previous study reporting that women undergoing laparoscopic hysterectomy typically fall within similar age ranges, often reflecting the prevalence of conditions such as fibroids and abnormal bleeding during these years [8]. In contrast, a nationwide analysis indicated that laparoscopic hysterectomy rates decline in women over 35 years, suggesting that older women may face more barriers to this surgical approach due to comorbidities or surgical risks [9].

The current study found that a significant proportion of patients had 3-4 children (47.9%), with only 2.6% having no children. This is consistent with other studies indicating that women who have undergone multiple pregnancies are more likely to seek surgical intervention for gynecological issues. For example, a large cohort study reported similar findings regarding parity distribution among patients undergoing laparoscopic hysterectomy, emphasizing that multiparity is common in this demographic [8,10-12].

In the current study, fibroids were the most common indication for surgery (42.7%), followed by abnormal uterine bleeding (35.0%). This is consistent with previous research, which has shown that fibroids are frequently cited as a primary reason for laparoscopic hysterectomy [13-15]. Another study highlighted that abnormal uterine bleeding is a prevalent indication, reinforcing the current study's findings [16].

The current study reported that most patients had uterine sizes corresponding to 11-12 weeks of gestation (31.6%) and 9-10 weeks (27.4%). Previous studies have documented similar trends, indicating that larger uterine sizes are standard among women undergoing hysterectomy due to conditions like fibroids [17]. A trend analysis revealed that more patients with larger uteri are now being treated laparoscopically, reflecting advancements in surgical techniques [17-22].

In the current study, 12 out of 117 patients required blood transfusions, indicating a relatively low incidence of significant hemorrhage during or after the procedure. This finding is consistent with other studies that have reported varying blood transfusion rates in laparoscopic hysterectomy cases. For example, a retrospective analysis indicated that the overall complication rate for laparoscopic hysterectomy was approximately 10%, with a specific blood transfusion rate of around 3% to 5% in many cases due to hemorrhagic complications [23]. Additionally, a study by Choudhary et al. highlighted that bladder injuries occurred in about 1.7% of patients, which often necessitated blood transfusions due to associated bleeding [5].

In contrast, the current study's higher transfusion rate may reflect a more complex case mix or variations in surgical technique among different practitioners. Moreover, a comprehensive review of laparoscopic and abdominal hysterectomies found that laparoscopic approaches generally have lower complication rates than abdominal surgeries [23]. This further supports the notion that while laparoscopic procedures are associated with some need for transfusions, they remain safer overall [23-25]. Overall, while the blood transfusion rate in the current study (10.3%) is higher than in some previous reports, it underscores the importance of careful surgical management and monitoring during laparoscopic hysterectomy procedures to minimize complications and ensure patient safety.

In the current study, 75.2% of patients underwent TLH, while 9.4% had TLH-BSO, and 15.4% had TLH combined with bilateral salpingectomy. These findings are consistent with other research indicating that TLH is the most commonly performed surgical technique for hysterectomy due to its minimally invasive nature and favorable outcomes [5]. For instance, a systematic review highlighted that TLH is associated with reduced postoperative pain, shorter recovery times, and lower complication rates than abdominal hysterectomy [26,27].

Moreover, a study by Choudhary et al. indicated that laparoscopic approaches, including TLH, are linked to lower rates of complications such as bladder and bowel injuries when performed by experienced surgeons [5]. This aligns with the current study's findings, suggesting that many patients can successfully undergo laparoscopic procedures without significant adverse events. Additionally, another study reported that laparoscopic hysterectomy has become increasingly preferred due to its advantages over traditional methods, such as reduced blood loss and shorter hospital stays [3]. The predominance of TLH in the current study underscores a growing trend toward minimally invasive surgical techniques in gynecological surgery.

In the current study, 6 out of 117 patients required conversion to open surgery, indicating a relatively low conversion rate. This finding is consistent with other research that has reported similar conversion rates in laparoscopic hysterectomy cases. For example, a systematic review indicated that conversion rates for laparoscopic hysterectomy typically range from 5% to 10%, depending on various factors such as the complexity of the case and the surgeon's experience [28]. A study by Choudhary et al. found that conversion rates were significantly lower among experienced surgeons, suggesting that surgical expertise is crucial in minimizing the need for conversion to open surgery. Their findings showed that as surgeons gain more experience with laparoscopic techniques, the incidence of conversions decreases, which aligns with the current study's results, indicating a manageable conversion rate. Moreover, another study highlighted that complications related to larger uterine sizes often necessitate conversions during laparoscopic procedures. In this context, the current study's findings suggest that while some patients may require conversion due to factors such as uterine size or intraoperative complications, the overall rate remains within acceptable limits compared to historical data [3,5,28,29].

In the current study, the most notable intraoperative complications included bladder injury (2.6%), bowel injury (0.9%), conversion to vaginal hysterectomy (0.9%), and conversion to laparotomy (3.4%). The overall low incidence of complications is consistent with findings from a systematic review that reported significant complications occurring in approximately 4.4% of laparoscopic hysterectomies, reinforcing that laparoscopic techniques are generally safe when performed by experienced surgeons. Moreover, a study by Choudhary et al. indicated that bladder injuries occurred in about 1.7% of patients undergoing laparoscopic hysterectomy, which aligns closely with the current study's findings [2,5]. This suggests that while bladder injuries can occur, they remain relatively rare and manageable with appropriate surgical techniques.

Additionally, other research has shown that bowel injuries during laparoscopic procedures are infrequent, with rates reported as low as 0.09% in some studies [3]. This is comparable to the current study's finding of a 0.9% incidence of bowel injury, suggesting that laparoscopic hysterectomy is associated with a low risk of this complication [3,5,29].

In the current study, the reported postoperative complications included intestinal obstruction (0.9%), paralytic ileus (0.9%), peritonitis (0.9%), and port-site infection (0.9%), with a notable majority of patients (96.6%) experiencing no complications at all. This low incidence of complications aligns with findings from a systematic review that reported overall postoperative complication rates for laparoscopic hysterectomy ranging from 2% to 6%, indicating that most patients recover without significant issues post-surgery [29]. A study by Choudhary et al. found that postoperative complications such as vault bleeding occurred in 2.75% of cases, slightly higher than the rates observed in the current study, but still reflecting a low overall complication rate. Moreover, they noted that intestinal obstruction and paralytic ileus were also infrequent but could occur in some patients, similar to the findings in the current study [5]. Additionally, another study indicated that urinary tract injuries and infections are common concerns following hysterectomy procedures, with rates varying significantly based on surgical technique and patient factors [3]. The current study's finding of port-site infection at 0.9% is consistent with the literature, suggesting that while infections can occur, they are generally manageable and infrequent in laparoscopic surgeries.

In the current study, the majority of patients (82.9%) required less than 48 hours stay in the hospital before discharge, which aligns with findings from a study that reported that 90% of patients undergoing laparoscopic hysterectomy were discharged within 24 hours post-surgery, with an average stay of approximately 22.9 hours [30]. This suggests that most institutions successfully implement protocols for rapid discharge following laparoscopic procedures; others may still adhere to more extended hospitalization periods. Additionally, the current study's discharge timing aligns with broader literature, indicating that recovery times can vary significantly based on surgical technique and patient factors. Moreover, a systematic review showed that recovery times for laparoscopic hysterectomy generally range from 2 to 6 weeks, depending on individual health factors and the complexity of the procedure. This variability in the recovery duration highlights the importance of tailored postoperative care and counseling to manage patient expectations regarding discharge and recovery [7,30].

Limitations

While the current study provides valuable insights into the outcomes of laparoscopic hysterectomy, several limitations must be acknowledged that may impact the generalizability and interpretation of the results:

Sample Size

The study included 117 patients, which may limit the statistical power to detect differences in outcomes or complications. A larger sample size could provide more robust data and enhance the reliability of the findings.

Single-Center Study

Conducting the study at a single institution may introduce biases related to specific surgical practices, patient demographics, and institutional protocols. Results may not be representative of broader populations or different healthcare settings.

Retrospective Design

If the study employed a retrospective design, it may be subject to selection bias and incomplete data. Retrospective studies often rely on existing medical records, which can lead to missing information or inconsistencies in data collection.

Variability in Surgical Techniques

The outcomes of laparoscopic hysterectomy can vary based on the surgeon’s experience and technique. This variability may affect complication rates and recovery times, making it challenging to draw definitive conclusions applicable to all surgical contexts.

Short Follow-Up Period

As the follow-up period was limited, long-term outcomes such as quality of life, recurrence of symptoms, and late complications may not have been adequately assessed. Longer follow-up is essential for understanding the full impact of surgical interventions.

Lack of a Control Group

Without a control group for comparison (e.g., patients undergoing abdominal hysterectomy), it is difficult to evaluate the relative effectiveness and safety of laparoscopic surgery against other surgical methods.

## Conclusions

In conclusion, this study highlights the effectiveness and safety profile of laparoscopic hysterectomy as a surgical option for managing gynecological conditions among women in their late reproductive years. The findings demonstrate that laparoscopic hysterectomy is associated with low intra-operative and postoperative complication rates, reinforcing its status as a preferred approach for conditions such as fibroids and abnormal uterine bleeding. Despite some patients requiring extended hospitalization, most experienced favorable outcomes with minimal complications, suggesting that laparoscopic techniques can be safely implemented in clinical practice. The demographic characteristics observed align with existing literature, confirming that women in their 40s and 50s are most affected by conditions warranting surgery.

However, it is essential to acknowledge this study's limitations, including its sample size, single-center design, and potential biases inherent in retrospective analyses. Future research should aim to include larger, prospective, randomized multicenteric studies/trials with more extended follow-up periods to validate these findings further and explore long-term outcomes associated with laparoscopic hysterectomy. Overall, this study contributes to the growing evidence supporting laparoscopic hysterectomy as a safe and effective treatment modality for women facing gynecological issues, ultimately enhancing patient care and surgical practices.
